# A stem cell proliferation burst forms new layers of P63 expressing suprabasal cells during zebrafish postembryonic epidermal development

**DOI:** 10.1242/bio.20136023

**Published:** 2013-09-16

**Authors:** Aida Guzman, Jose L. Ramos-Balderas, Samantha Carrillo-Rosas, Ernesto Maldonado

**Affiliations:** Departamento de Biología Celular y del Desarrollo, Instituto de Fisiología Celular, Universidad Nacional Autónoma de México, Circuito Exterior, Ciudad Universitaria, 04510, México, D.F., México

**Keywords:** Epidermal stem cells, Epidermal stratification, Postembryonic development, Zebrafish

## Abstract

Organ growth during development is a highly regulated process with both temporal and spatial constraints. Epidermal stratification is essential for skin growth and development. Although the zebrafish has been well studied, it is not known when and how epidermal stratification occurs. This is because beyond the first five days of development our knowledge is currently limited. We found that epidermal stratification in zebrafish begins when the larvae reach a standard length (SL) of 6 mm at approximately 25 days of age. Over the next four days (from a SL of 6 to 9 mm), epidermis thickness increases almost four-fold. This represents a sudden increase in organ size, since for the previous 20 days of development, the epidermis has been only two layers thick. This pattern is different from that observed in mammals that undergo continuous stratification from E14.5–E18.5. To study how stem cell proliferation gives rise to the new epidermal layers, we used a combination of markers: one for cell proliferation (proliferating cell nuclear-antigen PCNA) and one for epidermal stem cells (P63 transcription factor). We identified, throughout the stratification process, two different waves of cell division. Initially, the most basal epidermal cells divided and generated a subset of suprabasal cells (possibly transient-amplifying cells); within the next several days, the basal cells stopped dividing, and the suprabasal cells began proliferation, giving rise to most of the cell types in the new layers. This part of the process is similar to what has been recently found during epidermal stratification in mammals.

## Introduction

In vertebrates, the epidermis is maintained by epithelial stem cells that are capable of self-renewal and differentiation. These properties are established during development and are the result of a complex and precisely coordinated program in which stratification plays an essential role (Koster and Roop, 2007; Lechler and Fuchs, 2005). The zebrafish is a model organism suitable for the study of organ formation, including that of the skin (epidermis, dermis and hypodermis) (Beis and Stainier, 2006; Chang and Hwang, 2011; Rakers et al., 2010; Sire and Akimenko, 2004). Much is known about the development of the zebrafish epidermis during the first five days of embryonic development, in part due to studies of different zebrafish mutants. For example, in the lgl2 (large giant larvae) mutant, hemidesmosomes are absent, and epidermal cells undergo epithelial-mesenchymal transition; consequently, epidermal cells are detached and lost (Reischauer et al., 2009; Sonawane et al., 2009). Epidermal cell shedding also occurs in EpCam (Epithelial cell adhesion molecule) and Hai1a (a serine protease inhibitor of Matriptase1a) zebrafish mutants (Carney et al., 2007; Slanchev et al., 2009). Hai1a mutants also exhibit epidermal cell hyperproliferation (Carney et al., 2007), which has also been observed in the “*psoriasis*” mutant (Webb et al., 2008). Mutations that affect the formation of the basal membrane of the epidermis in zebrafish (*lama3*, *itga3*, *fras1*, *frem1-2*, *hmcn1*) produce a characteristic fin blistering phenotype and have been proposed as models for Fraser syndrome in humans (Carney et al., 2010; Feitosa et al., 2012). The development of certain structures that are ultimately located in the zebrafish epidermis but that originate elsewhere (such as peripheral sensory axons and neuromasts) has been studied (Hernández et al., 2007; O'Brien et al., 2012). Genetic tools for the study of epidermal development, such as the cytokeratin II promoter which drives epidermal expression (Wang et al., 2006) and a conditional cell ablation system that can be used to eliminate epidermal cells at any given time point (Chen et al., 2011), have also been designed.

During zebrafish development, the surface ectoderm is induced to an epidermal cell fate just a few hours after fertilization. By 24 hours post-fertilization (hpf), the epidermis (approximately 4 µm thick) is organized into two cell layers: an external enveloping layer and a basal layer (Le Guellec et al., 2004). For the next two to three weeks, the external layer protects the larvae from the environment while the fish grows. The basal layer produces large amounts of collagen to form the basal membrane and the primary dermal stroma, but the epidermis remains only two cell layers thick. It is not known when or how epidermal stratification takes place in zebrafish. Certain aspects of epidermal stratification were described in a very detailed electron microscopy study addressing zebrafish dermis development. The authors of this study reported that a third epidermal layer appeared by 15 days post-fertilization (dpf) and that four epidermal layers were visible by 26 dpf (Le Guellec et al., 2004). However, breeding conditions (population density, food quality, temperature) significantly affect growth rates (Parichy et al., 2009), and the developmental rate can therefore vary greatly from one laboratory to another. Thus, we believe that further analysis is required to understand how zebrafish epidermal stratification occurs and how it compares to the same process in other organisms.

In this work, we aim to identify the developmental stage at which epidermal stratification takes place in zebrafish. Taking advantage of a now well-established post-embryonic developmental staging series (Parichy et al., 2009), we describe when and how several critical cellular events in epidermal stratification take place.

## Results

### Epidermal thickening during postembryonic zebrafish development from a SL of 4.2 to 8.6 mm

**Fig. 1.**
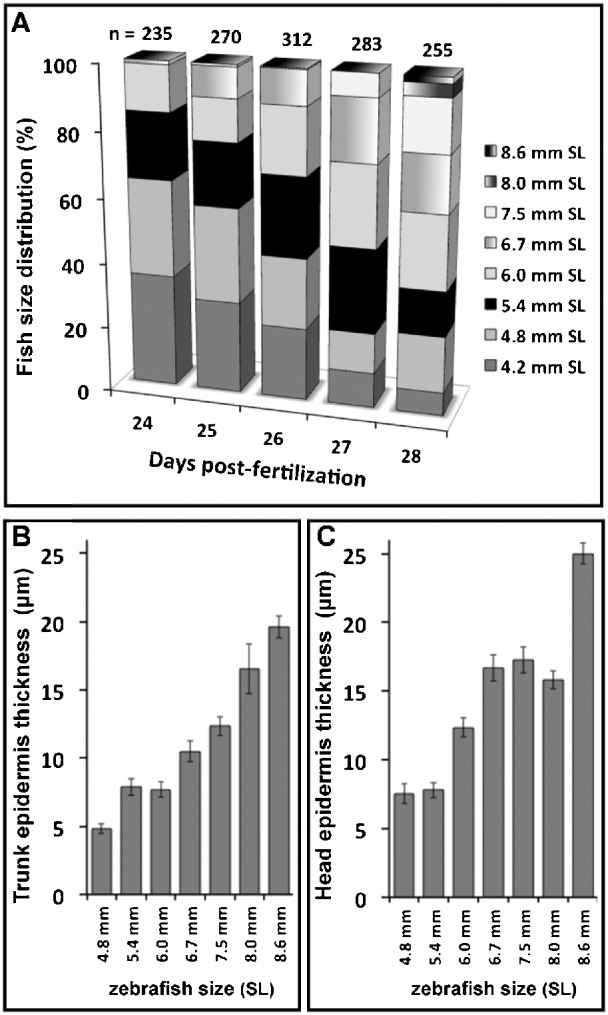
Distribution of zebrafish larvae size and epidermis thickness from 24 to 28 dpf. (A) Larvae were collected each day from 24 to 28 dpf, fixed and measured from the snout to the caudal peduncle (SL: Standard Length), then grouped into eight different classes according to their size (see Materials and Methods): SL of 4.2, 4.8, 5.4, 6.0, 6.7, 7.5, 8.0 and 8.6 mm. The size class distribution changes rapidly during this window, with SL above 7.5 mm appearing only at 27 and 28 dpf. (B) Epidermal thickening in the dorsal trunk region. (C) Epidermal thickening in the head area. (B and C) We measured the epidermal thicknesses in histological sections taken from fish with a SL of 4.8 mm to 8.6 mm and found a nearly fourfold increase in epidermal dimensions. Epidermal thickness was measured in fourteen larvae from each size class, and the mean values were used to build class histograms. Error bars represent the standard error of the mean.

In preparation for this work, we analyzed the epidermis in histological sections from larvae at 10, 15, 20, 25 and 30 dpf (data not shown); epidermal changes were only observed after 25 dpf. For this reason, we chose to study the epidermis during a window surrounding this period of time. Our first aim was to look for epidermal thickening during postembryonic zebrafish development; with that in mind, we collected and studied larvae between 24 and 28 dpf. Because, during postembryonic stages, size is a better indicator of developmental progress than age (Parichy et al., 2009), we measured the antero-posterior (SL) length from the snout to the caudal peduncle and grouped the collected larvae into eight different size classes with mean SL values of 4.2, 4.8, 5.4, 6.0, 6.7, 7.5, 8.0 and 8.6 mm (see Materials and Methods). We observed that zebrafish larvae double in SL from days 24 to 28 of development, as reflected by the different size class distributions found on each day (Fig. 1A). While the larvae with a SL of 4.2 to 5.4 mm represent 84% of the population at 24 dpf, only 38% of the population falls into these classes at 28 dpf. Larvae with a SL greater than 7.5 mm could only be found on days 27 and 28. We wanted to determine how this size increase was related to epidermal growth. Therefore, we measured epidermal thickness in larvae with a SL of 4.8 to 8.6 mm. We found that, during these days of development, the epidermis grew at a constant rate, increasing its thickness fourfold (Fig. 1B,C). The same pattern was observed in the dorsal trunk region (Fig. 1B) and in the head area (Fig. 1C) where the epidermis appeared to thicken at a similar rate.

### Epidermal stratification starts when larvae attain a SL of 5.4 and 6 mm

**Fig. 2.**
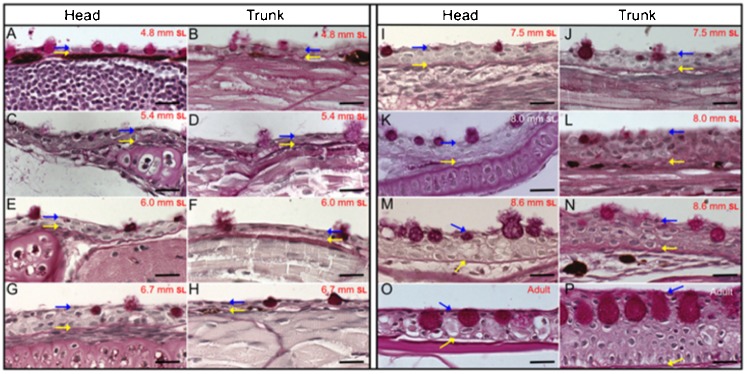
Histological longitudinal sections and periodic acid-Schiff staining of juvenile fish with a SL of 4.8 mm to 8.6 mm and from adult zebrafish. (A, C, E, G, I, K, M and O) Sections from the head region. (B, D, F, H, J, L, N and P) Sections from the dorsal trunk area. (A, B), (C, D), (E, F), (G, H), (I, J), (K, L) and (M, N) are from fish with a SL of 4.8 mm, 5.4 mm, 6 mm, 6.7 mm, 7.5 mm, 8 mm and 8.6 mm, respectively. (O, P) Sections of the head and dorsal trunk from adult fish. Blue arrows label the topmost limit of the epidermis and yellow arrows label the basal membrane, which is specifically labeled by PAS. (C–F) Epidermal stratification and thickening was initiated when larvae attained a SL of 5.4 and 6 mm. (G–N) When larvae reached a SL of 6.7–8. 6 mm, stratification notably thickened the epidermis. During this time, the mucous cells undergo a considerable increase in size, which might give the impression that these images were not taken at the same magnification; however, all images shown here were obtained with a 63× oil objective (Zeiss). The large spaces visible between the skin and other tissues may be artifactual. Bars are 20 µm in length.

For the analysis of zebrafish epidermal stratification, histological sections were prepared from larvae with a SL of 4.8 to 8.6 mm. These sections were stained using the periodic acid–Schiff (PAS) method, which labels the basal membrane that separates the epidermal and dermal layers. Observations of both the head and dorsal trunk regions indicated that the thickening of the epidermis observed during this developmental window results from an increase in the number of cell layers (Fig. 2). In the head area, the epidermis began to thicken when larvae reached a SL of 6 mm, while, in the dorsal trunk, thickening was observed at a SL of 6.7 mm (Fig. 2E–H, Fig. 1B,C). Mucous cells were also labeled with the PAS stain. We noted that, as the epidermis grew, the mucous cells also became considerably larger (Fig. 2). By the 8.6-mm-SL developmental stage, the epidermis was four to six cell layers thick (Fig. 2M,N). When comparing the postembryonic larval epidermis (Fig. 2A–N) with the adult epidermis (Fig. 2O,P), it is obvious that the epidermis in the head reaches its maximum thickness (which, in adults, measures on average 20 µm) during the postembryonic development stages studied here (SL of 4.8–8.6 mm). In contrast, the dorsal trunk epidermis (which is close to 25 µm thick in larvae with a SL of by 8.6 mm) remains far from its maximum thickness of 40–60 µm (Fig. 2P). The epidermis in the ventral abdomen of adult fish is very thin, with an average thickness of 10 µm (data not shown).

### Cell proliferation during epidermal stratification

**Fig. 3.**
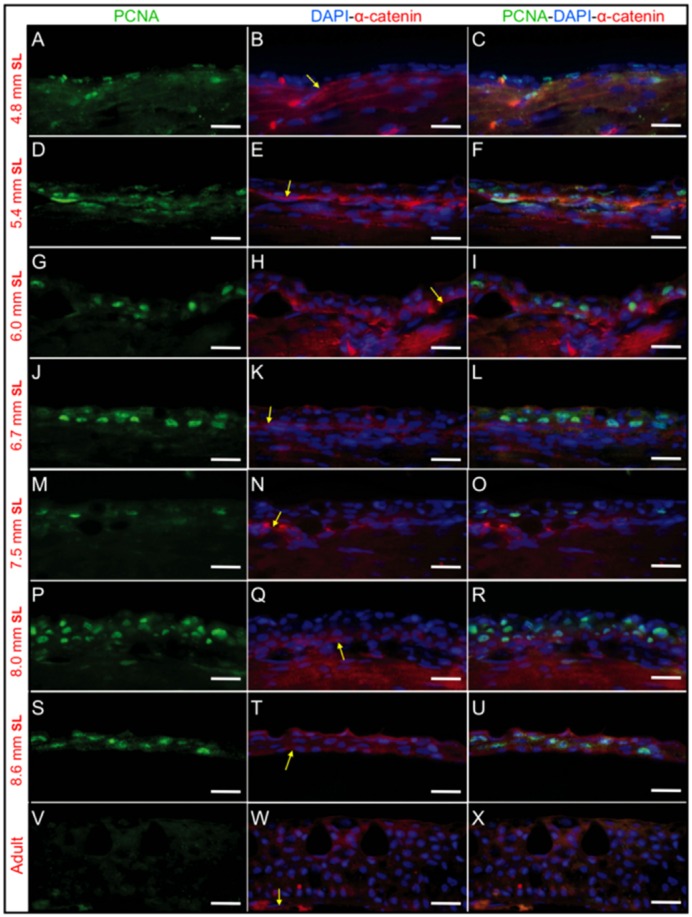
PCNA and α-catenin immunofluorescence staining reveals cell proliferation during epidermal stratification in the dorsal trunk area. Anti-PCNA labels proliferating cells, while anti-α-catenin labels both the epidermis and dermis but is concentrated in the basal membrane, which allows the discrimination of these two regions. (A–C), (D–F), (G–I), (J–L), (M–O), (P–R) and (S–U) show immunofluorescence staining from longitudinal sections of larvae with a SL of 4.8 mm, 5.4 mm, 6 mm, 6.7 mm, 7.5 mm, 8 mm and 8.6 mm SL, respectively. PCNA labeling is observed specifically in basal layer cells from SL 4.8–5.4 mm larvae (A–F), but later is observed in suprabasal layers (compare C, L and R). (V–X) are immunostained longitudinal sections from adult fish; no PCNA staining can be observed in this representative region. In some samples (like S–U) the epidermis get separated from the dermis but this is artifactual. Yellow arrows mark the epidermal basal membrane where α-catenin labeling concentrates. Bar length is 20 µm.

**Fig. 4.**
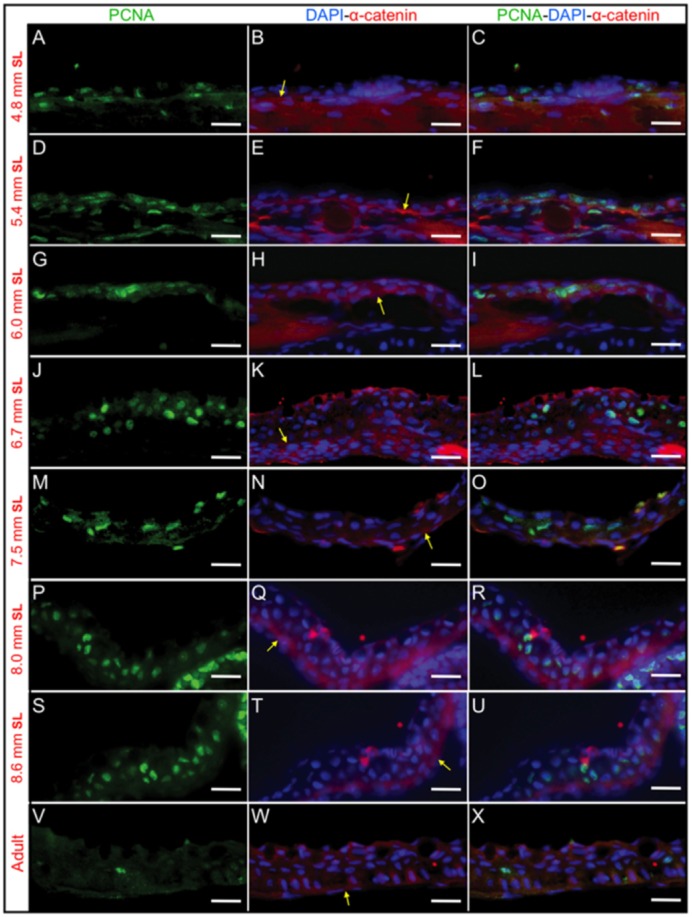
PCNA and α-catenin immunostaining to show cell proliferation during epidermal stratification in the head area. Anti-PCNA labels cells in proliferation and anti-α-catenin labels the basal membrane that separates the epidermis and dermis regions. (A–C), (D–F), (G–I), (J–L), (M–O), (P–R) and (S–U) show immunostained longitudinal sections of larvae with a SL of 4.8 mm, 5.4 mm, 6 mm, 6.7 mm, 7.5 mm, 8 mm and 8.6 mm, respectively. (V–X) show immunostained longitudinal sections in adult fish. (A, D, G, J, M, P, S and V) PCNA immunostaining. (B, E, H, K, N, Q, T and W) Anti-α-catenin immunofluorescence merged with DAPI staining to label the nuclei. (C, F, I, L, O, R, U and X) Merged images of PCNA, α-catenin and DAPI staining. In some samples (like M–O) the epidermis get separated from the dermis, however this is artifactual. Yellow arrows mark the epidermal basal membrane where α-catenin labeling is abundant. Bar length is 20 µm.

To determine whether stratification is a consequence of cell proliferation, we carried out immunostaining in histological sections from larvae during the postembryonic development period. Anti-PCNA was used to label the nuclei of cells that are on the point of entering cell division. At the same time, we used an anti-α-catenin antibody to label the cell membranes of epidermal cells; α-catenin is specifically enriched at the zone of contact with the basal membrane of the epidermis. As expected, we observed a high degree of proliferation in the epidermis in larvae with a SL between 4.8 mm and 8.6 mm in both the dorsal trunk area (Fig. 3A–U) and in the head region (Fig. 4A–U). While some proliferation was observed in the dermis area, we were able to distinguish epidermal from dermal proliferation (as an example, see Fig. 3D–F) using the α-catenin labeling. Immediately prior to a SL of 4.8 mm, and at the initial stratification developmental stages (SL of 5.4–6 mm), most of the proliferating cells appeared to be in contact with the epidermal basal membrane (Fig. 3A–I, Fig. 4A–I); however, as stratification progressed, the PCNA-positive cells became more broadly distributed in the recently formed suprabasal layers (Fig. 3J–U, Fig. 4J–U). In the adult epidermis, PCNA labeling was less abundant (Fig. 3V–X, Fig. 4V–X). We observed that in larvae with a SL of 4.8–5.4 mm, the nuclei of the proliferating epidermal cells were flat and elongated; however, subsequently (at a SL of 6–8.6 mm SL), the nuclei in the proliferating cells were more rounded.

### Most cells proliferating during epidermal stratification express P63 protein

**Fig. 5.**
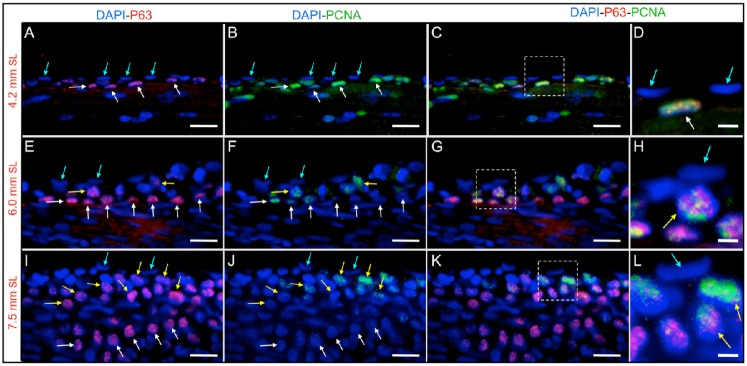
Zebrafish epidermal stratification as a consequence of the proliferation of P63-expressing cells. (A–D) Longitudinal sections from larvae with a SL of 4.2 mm. (E–H) Longitudinal sections of larvae with a SL of 6 mm. (I–L) Longitudinal sections of larvae with a SL of 7.5 mm. (A, E and I) P63 and DAPI labeling. (B, F and J) PCNA and DAPI immunofluorescence. (C, G and K) Merged P63, PCNA and DAPI stainings; the colocalization of P63 and DAPI appears magenta, and the colocalization of P63 and PCNA appears yellow. (D, H and L) Higher magnification images of the regions marked by squares in (C, G and H), respectively. Prior to the initiation of stratification (SL of 4.2 mm), basal cells (P63 positive) express the proliferation marker PCNA; later, for a SL of 6 mm, new suprabasal cells appear, also exhibiting P63 and PCNA co-labeling. For a SL of 7.5 mm SL, suprabasal cells (P63 positive) are abundant, and only suprabasal cells show PCNA labeling. Blue arrows label cells from the external epidermal layer, yellow arrows mark suprabasal cells at intermediate layers that are positive for PCNA and P63 expression, white arrows label P63 positive cells from the basal layer. Bars are 20 µm in length, except in (D, H and L), where they are 5 µm in length.

Prior to the initiation of stratification, only two epidermal cell layers are present (the basal and external layers), and these have different morphological and functional characteristics (Le Guellec et al., 2004). Only basal cells express P63 (a P53 homolog), which is required to maintain the regenerative capacity of the epithelial stem cells (Fuchs, 2009; Hsu and Fuchs, 2012; Koster et al., 2004; Yang and McKeon, 2000). To determine whether the basal cells are actively proliferating during epidermal stratification, we combined the anti-PCNA and anti-P63 antibodies (Fig. 5); both PCNA and P63 are expressed in the cell nuclei. We found that, in larvae with a SL of 4.2 mm, PCNA-expressing cells also expressed P63 (Fig. 5A–D; supplementary material Fig. S1). We believe these are basal cells, as the only epidermal cells that express P63 are located within the basal cell layer (Bakkers et al., 2002; Koster et al., 2004; Lee and Kimelman, 2002). Later, when larvae reached a SL of 6–7.5 mm, a reduction in the number of basal cells expressing PCNA was observed, even though P63 expression was maintained (Fig. 5E–L; supplementary material Fig. S1).

**Fig. 6.**
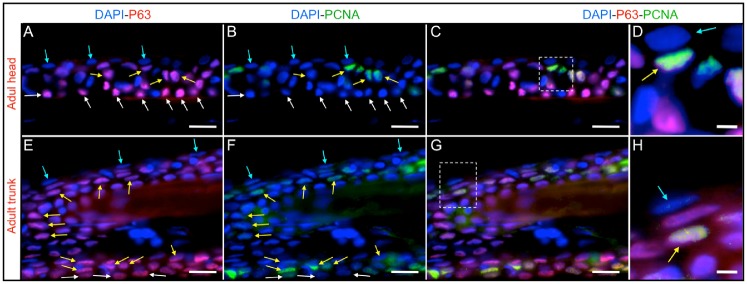
PCNA and P63 immunofluorescence in the adult zebrafish epidermis. (A–D) Head region. (E–H) Dorsal trunk area, in a region that contains only scales. (A and E) DAPI and P63 labeling. (B and F) DAPI and PCNA immunofluorescence. (C and G) Merged P63, PCNA and DAPI immunostainings. (D and H) Higher magnification images of the regions marked by squares in (C and G), respectively. Suprabasal P63-positive cells persist in adulthood and remain actively proliferating. Blue arrows mark cells from the external epidermal layer, yellow arrows label suprabasal cells at intermediate layers that are positive for PCNA and P63 expression, white arrows mark P63 positive cells from the basal layer. Bars are 20 µm in length, except in (D, H and L) where they are 5 µm in length.

At this point (SL 6 mm), we observed the appearance of a suprabasal or intermediate layer of PCNA- and P63-expressing cells that had rounded nuclei, in contrast to the flat nuclei characteristic of basal cells (Fig. 5E–H). Stratification appeared to occur rapidly when larvae reached a SL of 7.5 mm, by this developmental stage, a notable increase in the number of suprabasal P63-expressing cells was observed (Fig. 5I–L) in the intermediate epidermal layers. PCNA expression had completely disappeared from the more basal layers, which remained positive for P63 expression. From this stage on, most P63-expressing cells had rounded nuclei (basal and intermediate). In adult fish, we only observed PCNA labeling in P63-positive suprabasal cells in the head (Fig. 6A–D) as in the trunk area (Fig. 6E–H). It is worth mentioning that PCNA was never observed within the topmost layer of external cells.

## Discussion

We combined histological and immunofluorescence analyses to determine the developmental stage at which epidermal stratification occurs in zebrafish; we found that this event occurs during postembryonic development, between 24 to 28 dpf. Because fish developmental growth rates strongly depend on breeding conditions (e.g., temperature, density, food quality) and therefore could vary among research facilities, we chose to use a more precise staging system proposed by other authors (Parichy et al., 2009; Sire et al., 1997) using larvae length as an index of development instead of dpf. Epidermal stratification begins when the SL of the larvae is between 5.4 and 6 mm, and the average rate of thickening is approximately 4.7 µm for every 1-mm increase in SL; subsequently, the epidermis grows to an approximate thickness of 20 µm in the adult head area and of 40–60 µm in the adult dorsal trunk region. Considering that there are only two epidermal layers of cells (4–5 µm thick) for approximately 25 days, the nearly fourfold thickening that occurs in the epidermis within five days represents a sudden and rapid increase in organ size. Other regions of the zebrafish skin are also thickening at this time; it has been reported that the deepest dermis layer, known as the “stratum compactum”, grows from a width of 2 µm in larvae with a SL of 7.5 mm to a width of 5 µm in larvae with a SL of 9 mm (Sire et al., 1997). It is worth mentioning that epidermal stratification precedes scale formation, which has been described to take place when larvae attain a SL between 8 to 10 mm (Parichy et al., 2009; Sire and Akimenko, 2004; Sire et al., 1997).

Interestingly, the approximately 25-day gap between epidermal specification (formation of the first two layers of cells) and stratification (formation of all other epidermal layers) does not occur in mammals. For example, epidermal specification begins at E8.5 in mice, and stratification takes place immediately thereafter, culminating with several layers of differentiated epidermal cells at E18.5 (Koster and Roop, 2007). This heterochronic change may be the consequence of adaptation while preparing (during development) the epidermis for the different environments in which these organisms live. It would be interesting to identify the molecular mechanisms that regulate this “stratification delay”. For example, in mammals, a certain balance between PP63 (the phosphorylated form of P63) and P63 (non-phosphorylated form) is required to initiate epidermal stratification (Koster et al., 2004); it may be that the appropriate balance between these two isoforms is not achieved until the fish grows to a certain size (5.4–6 mm SL). PP63 phosphorylation is also associated with certain events in stratification (Suzuki and Senoo, 2012). It would be very useful to produce specific zebrafish PP63 antibodies for use in future studies.

**Fig. 7.**
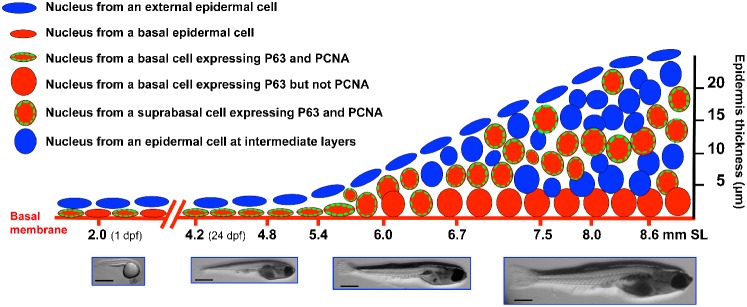
Model for epidermal stratification during postembryonic development in zebrafish. This diagram shows the labeling in the nuclei from epidermal cells, through different postembryonic developmental stages, as was observed in this work. By 1 dpf the embryo has an approximate size of 2 mm in length, the epidermis is formed by two cell layers, the external layer and the basal layer. The basal layer is known to express the P63 transcription factor (a known marker for epidermal stem cells). Just before epidermal stratification initiates (SL 4.2–4.8 mm) there are high levels of proliferation in the basal layer. When the fish reach a size of SL 5.4–6 mm, epidermal stratifications begins. At this point the nuclei of basal cells became rounded and these cells reduce proliferation. A new layer of suprabasal cells appears which also express P63 and shows an active cell proliferation. At stages of SL 8.0–8.6 the epidermis is 5–8 layers thick (depending of the body region) and suprabasal cells are still proliferative while basal cells have completely stop proliferation. It is possible that suprabasal cells are “transient-amplifying cells”. Bars are 1 mm in length.

We observed a change in the PCNA labeling pattern during epidermal stratification. When the larvae had reached a SL of 4.8–5.4 mm, PCNA-labeled cells were present only among the cells in contact with the basal membrane (labeled with the α-catenin antibody); however, when the larvae had grown to a SL of 6–8.6 mm, PCNA-positive cells were now widespread in the recently formed epidermal layers. In order to identify which epidermal cells proliferate during stratification, we specifically labeled the zebrafish basal layer with an antibody that marks P63 expression. P63 is a P53 homolog that is regulated by BMP and is essential for the development of the epidermal ectoderm. P63 has been used as a marker for epidermal stem cells; in mammals, epidermal stratification is associated with the proliferation of stem cells (Hsu and Fuchs, 2012; Koster et al., 2007; Koster et al., 2004; Lander et al., 2012). Our observations show an interesting dynamic between epidermal P63 expression and cell proliferation during the stratification process in zebrafish (Fig. 7).

First, during the initial steps of stratification (SL of 5.4–6 mm) when there are only two layers of cells (basal and external), PCNA and P63 co-labeling is restricted to basal cells. This has also been observed during the first days of development, though PCNA expression is less abundant (Horng et al., 2009). In zebrafish early developmental stages, P63 is expressed around 5 hpf and is required for dorsoventral patterning and the formation of the epidermis and limbs during early development. During the earliest stages of development, P63 is distributed throughout the cytoplasm; however, by 20 hpf, it is localized in the cell nuclei (Bakkers et al., 2002; Lee and Kimelman, 2002). *In vitro* studies have shown that P63 induces proliferation and prevents premature differentiation in mammalian basal keratinocytes, giving rise to stratification (King et al., 2006) (Fig. 7).

Second, starting at the 6-mm-SL stage, suprabasal cells expressing PCNA and P63 appear, accompanied by a reduction in the number of basal cells showing PCNA labeling. The same phenomenon has been observed in mice between E13.5 and E14.5 (Koster and Roop, 2007); in this case, stem cells coexist within the basal layer with a set of their daughter cells known as transit-amplifying cells that later move suprabasally to initiate terminal differentiation (Koster et al., 2007; Suzuki and Senoo, 2012). This possibly means that not all specific zebrafish epidermal cell types are generated by the basal stem cells but that there is a relay in which suprabasal progenitors or transit-amplifying cells participate. One example of transit-amplifying cells are the “intermediate neural progenitors”, which have a more restricted proliferative potential than stem cells, but can still produce an enormous amount and diversity of neural cells during development (Kriegstein and Alvarez-Buylla, 2009; Viktorin et al., 2013). It was recently reported that P63 is phosphorylated in suprabasal cells but not in basal cells (Suzuki and Senoo, 2012); it may be possible to use a phospho-specific antibody to monitor the dynamics of epidermal transit-amplifying cell formation during zebrafish development (Fig. 7).

Third, multiple layers of cells, form the zebrafish epidermis in the larvae with a SL of 7.5 mm. The suprabasal or transit-amplifying P63-expressing cells are abundant at this stage, but only some of these cells are proliferating (PCNA positive). At this point, basal cells no longer express PCNA, and their nuclei are now rounded. This pattern of expression is similar to the pattern observed in the adult fish epidermis, though PCNA expression is less abundant in the adult. P63-expressing cells in both the basal and suprabasal layers have also been recently found in the adult human epidermis (Suzuki and Senoo, 2012) (Fig. 7).

In this work we provide the structural framework for future molecular mechanistic studies in zebrafish epidermal stratification. In mammals, epidermal stratification is associated with a change in spindle orientation (Muroyama and Lechler, 2012) it will particularly interesting to look now for asymmetric cell divisions and the associated molecular regulatory gene network, during zebrafish epidermal development.

## Materials and Methods

### Fish husbandry

Wild-type zebrafish (*Danio rerio*) embryos were obtained from natural crosses of fish with the TAB-14/WIK genetic background. The TAB-14 strain was kindly donated to us by Professor Nancy Hopkins from MIT, while the WIK strain was obtained from the Zebrafish International Resource Center (ZIRC). Adult zebrafish were maintained in a recirculation system (Aquatic Habitats) with a constant pH, temperature and dark-light cycle (Trevarrow, 2004). The food consisted of harvested nauplii larvae mixed with macerated Tetramin Pro (Tetra). Zebrafish embryos obtained by natural crosses were placed in Petri dishes (60 embryos per plate) and maintained at 28.5°C for 5 days. At 6 dpf, the larvae were moved to 200-ml glass beakers at a density of 30 larvae per beaker in 100 ml of fish water (250 mg/L Instant Ocean, 4 mg/L NaHCO_3_, pH 7); the larvae were fed three times a day with 10 ml of paramecia (100–150 cells per ml). Water was changed every other day. At 13 dpf, three drops of brine shrimp were added to each meal, the temperature was maintained at 21–22°C. Sixteen dpf; larvae were transferred to mesh-bottom cylinders (height, 7.5 cm; diameter, 8.2 cm) fitted in 3-L tanks that were placed in a recirculating system (Nursery 3; Aquatic Habitats) at 26°C and maintained at a density of 30 individuals per cylinder. In these cylinders, the feed was concentrated within a smaller volume, allowing for appropriate fish growth. At this time, the diet was changed to 5 ml of brine shrimp mixed with macerated Tetramin-Pro (Tetra), fed three times per day. Twenty-three dpf; the larvae were transferred to 1-L tanks (with a 750 micron baby baffle) at a density of 30 larvae per tank and fed the same diet. All procedures performed with animals were approved by the Office of Laboratory Animal Welfare (OLAW) of the United States National Institutes of Health (NIH), approval #A5281-01.

### Larval fish measurements

Zebrafish larvae were collected each day from 24 to 28 dpf, euthanized in ice-cold 0.025% tricaine (3-aminobenzoic acid methyl ester from Sigma) and fixed in 4% paraformaldehyde at pH 7.4 for 12 h. After fixation, the medium was replaced with PBS (pH 7.4), and measurements were performed in a Stemi-SV11 stereomicroscope (Zeiss) equipped with a 10× eyepiece reticle and previously calibrated using a stage micrometer. Standard Length (SL) measurements were made from the snout (most anterior point of the head excluding the lower jaw) to the caudal peduncle (most posterior region of the body where caudal fins rays insert), as described by Parichy et al. (Parichy et al., 2009). To group the fish according to their size, each larva was placed in one of eight tubes (labeled 4.0, 5.0, 5.5, 6.0, 6.5, 7.5, 8.0 and 8.5 mm SL) reflecting the value closest to its measured size. Then, using the actual measurements for the larvae in each tube, we calculated the mean value and re-named each group according to the mean. Thus, eight size classes were created: 4.2, 4.8, 5.4, 6.0, 6.7, 7.5, 8.0 and 8.6 mm SL. We believe these classes better reflect the sizes of the zebrafish during growth. We examined each size class for reported traits and characteristics of each size or developmental stage and found a satisfactory correlation between our results and those reported elsewhere (Parichy et al., 2009). Measurements of the epidermal thickness were made with the AxioVision software (Zeiss) from histological preparations digitalized in a pre-calibrated AxioImager microscope. All images were taken with the same exposure time for all larvae. Some images were enhanced using Adobe Photoshop, but all images in the same figure were placed side by side in the same layer prior to any enhancements or modifications to avoid biased comparisons.

### Histological sectioning and periodic acid-Schiff staining

After measurements were taken, the larvae were post-fixed in NaOAc-formaldehyde-glycerine (1.8% NaOAc, 3.6% formaldehyde and 10% glycerine) for 12 h, washed with PBS (pH 7.4), gradually dehydrated with ethanol (60, 70, 80, 96 and 100%) for 20 min each and cleared with xylene (JT Baker) twice for 20 min each. At this point, samples were embedded in melted paraffin wax (Leica), and 5-µm sections were prepared in a rotatory microtome (Leica RM2145). To label the basal membrane for our analysis of the epidermis, we used periodic acid-Schiff (PAS) staining, which is specific for glycoproteins. Briefly, sections were incubated in 0.5% periodic acid for 10 min, in Schiff solution (0.4% Basic Fuchsin, 0.09 N HCl and 0.9% K_2_S_2_O_5_) for another 10 min, washed in water and post-stained with Gill no. 1 hematoxylin (Sigma).

### Immunofluorescence and antibodies

Histological sections were deparaffinized, rehydrated and treated for antigen recovery. Blocking and permeabilization were performed simultaneously (0.1% Tween and 2% albumin), and the sections were incubated first with the indicated primary antibody at 4°C for 12 h then with Alexa Fluor-conjugated secondary antibodies for 2 h (Invitrogen). The anti-α-catenin antibody (ab-49105) was obtained from Abcam, while the anti-PCNA (sc-7907) and anti-P63 (sc-8431 clone H-137) antibodies were obtained from Santa Cruz Biotechnology, Inc. Multiple isoforms of P63 exist in zebrafish (as in other organisms); the major isoforms are known as ΔNP63 and TAP63. According to the company that provides the antibody, this was prepared against the Human ΔNP63 isoform (residues 15–151), however it is not known if the same antibody also binds to the TAP63 or any other isoform. A similar antibody (clone 4A4, that is no longer produced) was used in one study (Lee and Kimelman, 2002), the authors observed that morpholino based inhibition of ΔNP63 eliminated the reactivity of the 4A4 antibody, therefore it is possible that the predominant isoform expressed in the zebrafish skin is ΔNP63. For that reason, along the text, we used the term P63 when referring to the binding of the sc-8431 antibody in epidermal cells. Fluorescence images were obtained with an AxioImager microscope using an MRc AxioCam camera and AxioVision 4.8 software (Zeiss).

### Statistical analysis

All experiments were performed at least three times. Data are given as the mean ±SD. *P*>0.05 was considered significant.

## Supplementary Material

Supplementary Material
