# Supplementary material for: A stem cell proliferation burst forms new layers of P63 expressing suprabasal cells during zebrafish postembryonic epidermal development
Source: Biol Open. 2013 Sep 16;2(11):1179–86. doi: 10.1242/bio.20136023 (PMC3828764; doi:10.1242/bio.20136023)
Supplement: Supplementary Material [file supp_2_11_1179__index.html]

A stem cell proliferation burst forms new layers of P63 expressing suprabasal cells during zebrafish postembryonic epidermal development — A stem cell proliferation burst forms new layers of P63 expressing suprabasal cells during zebrafish postembryonic epidermal development — Supplementary Material 

# A stem cell proliferation burst forms new layers of P63 expressing suprabasal cells during zebrafish postembryonic epidermal development

## bio.20136023 Supplementary Material

**Files in this Data Supplement:**

- Supplementary Material - Aida Guzman et al. doi: 10.1242/bio.20136023
