# Supplementary material for: A stem cell proliferation burst forms new layers of P63 expressing suprabasal cells during zebrafish postembryonic epidermal development
Source: Biol Open. 2013 Sep 16;2(11):1179–86. doi: 10.1242/bio.20136023 (PMC3828764; doi:10.1242/bio.20136023)
Supplement: Supplementary Material [file supp_bio.20136023_bio.20136023-s1.pdf]

Biology Open

## Supplementary Material

**Aida Guzman et al. doi: 10.1242/bio.20136023**

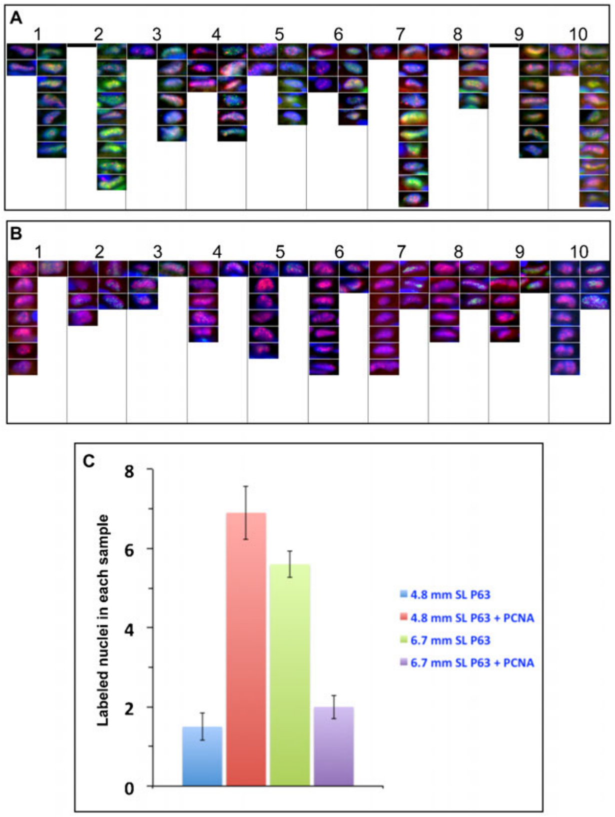


**Fig. S1. Quantification of the PCNA changes in expression from basal cells during epidermis stratification.** (A) For ten samples of the 4.8 mm SL epidermis co-immunostained for P63 and for PCNA, nuclei that only express P63 are shown in the columns to the left and the ones with both P63 and PCNA expression, in the columns to the right. (B) Also, in ten samples of 6.7 mm SL epidermis co-immunostained for P63 and for PCNA, the nuclei that are only positive for P63 are shown in the left columns, while those positive for both P63 and PCNA in the right columns. The 4.8 mm SL basal cells nuclei are more flat in shape than those from the 6.7 mm SL. PCNA expression in 6.7 mm SL epidermal basal cells was not only less frequent but also less intense.
(C) Histogram showing that PCNA labeling in the nuclei from epidermal basal cells decrease from larvae fish 4.8 mm SL to 6.7 mm SL larvae. Error bars represent the standard error of the mean.
